# Functionalization of Carbon Nanofibers with an Aromatic Diamine: Toward a Simple Electrochemical-Based Sensing Platform for the Selective Sensing of Glucose

**DOI:** 10.1021/acsomega.4c00525

**Published:** 2024-06-11

**Authors:** Angelo Ferlazzo, Consuelo Celesti, Daniela Iannazzo, Claudio Ampelli, Daniele Giusi, Veronica Costantino, Giovanni Neri

**Affiliations:** † Department of Chemical Sciences, University of Catania, Viale Andrea Doria, 6, I-95125 Catania, Italy; ‡ Department of Engineering, University of Messina, Contrada Di Dio, I-98166 Messina, Italy; § Department of Chemical, Biological, Pharmaceutical and Environmental Sciences (ChiBioFarAm), University of Messina and INSTM, Via F. Stagno d’Alcontres 31, I-98166 Messina, Italy

## Abstract

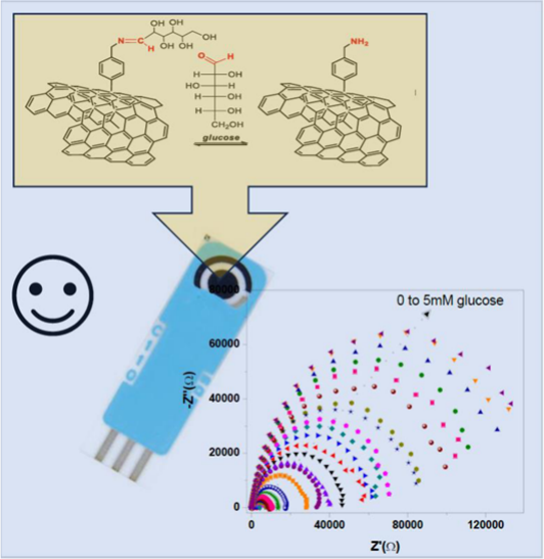

Despite a variety of glucose sensors being available today, the development of nonenzymatic devices for the determination of this biologically relevant analyte is still of particular interest in several applicative sectors. Here, we report the development of an impedimetric, enzyme-free electrochemical glucose sensor based on carbon nanofibers (CNFs) functionalized with an aromatic diamine via a simple wet chemistry functionalization. The electrochemical performance of the chemically modified carbon-based screen-printed electrodes (SPCEs) was evaluated by electrical impedance spectroscopy (EIS), demonstrating a high selectivity of the sensor for glucose with respect to other sugars, such as fructose and sucrose. The sensing parameters to obtain a reliable calibration curve and the selective glucose sensing mechanism are discussed here, highlighting the performance of this novel electrochemical sensor for the selective sensing of this important analyte. Two linear trends were noted, one at low concentrations (0–1200 μM) and the other from 1200 to 5000 μM. The limit of detection (LOD), calculated as the (standard error/slope)*3.3, was 18.64 μM. The results of this study highlight the performance of the developed novel electrochemical sensor for the selective sensing of glucose.

## 1 Introduction

Glucose is an aldose monosaccharide that plays a fundamental role in the processes of photosynthesis and respiration, also serving as an energy reserve and a metabolic fuel of mammalian cells.^1^ Glucose monitoring allows the detection of changes in blood concentrations in response to diet, exercise, medications, and disease processes associated with glucose fluctuations, such as diabetes mellitus.^2,3^ High or low blood glucose levels can result in life-threatening complications that include heart and kidney diseases, retinopathy, decreased quality of life, expensive surgeries, or even death.^4^ Furthermore, as a component of more complex structures such as polysaccharides and glucosides, glucose plays an important role in energy storage and as components of plant cell walls.^1,5^ Due to its pivotal role in biological mechanisms, the quantification of this sugar is of great interest not only for the diagnosis and control of human diseases, including diabetes, but also for the monitoring of mammalian cell growth and for the analysis of agricultural products to ensure food quality and safety.^6,7^ Despite a variety of glucose sensor devices being available, also being on the market from a long time, the development of electrochemical sensors for the determination of glucose is still investigated today.^8,9^ Enzymatic sensors for the determination of glucose are widely used in the clinical field,^10−12^ but they have several limitations^13^ as the measurements should be carried in physiological conditions of temperature and pH to avoid the degradation of the enzyme recognition layer.^14^ Therefore, the development of sensors for glucose determination without the use of enzymes is of particular interest, especially in other applicative sectors such as the agrifood and fermentation industry.^15^ Many carbon-based materials, such as carbon nanotubes (CNTs), carbon nanofibers, graphene, graphene oxide (GO), and graphene quantum dots (GQDs) have been exploited to improve the glucose sensing performances.^14−22^ The use of organic molecules to functionalize carbon materials is a widely applied method for the development of new electrochemical sensors.^21^ In particular, carbon materials functionalized with boronic acid have been reported as efficient glucose sensors.^14,23−25^

Among the different classes of carbon-based nanomaterials, CNFs have been investigated for the development of biosensors due to their high conductivity that allows an efficient electron transfer to the electrode surface.^25^ These nanomaterials are similar in structure and properties to CNTs, but are low-cost, easier to produce, and provide improved functionalities.^26^ Nonenzymatic electrochemical sensors based on CNFs reported so far are mainly constituted by metal and metal oxide nanoparticles loaded on their high specific surface area to enhance the electrocatalytic activity of the nanocomposites and the performance of the biosensors.^27^

In this study, we focused our attention on the development of an enzyme-free electrochemical glucose sensor based on CNFs, taking advantage of the chemical reactivity of π-electrons of the external graphite layer,^28^ using an aromatic diamine. This allowed them to bind an aromatic and highly conjugated system covalently on CNFs, leaving a free amino functional group able to selectively interact with glucose.

Fundamental points of this work highlighted are a simple synthesis procedure to obtain a sensitive material for glucose not yet reported in the literature and its use for the selective impedimetric glucose sensing by electrical impedance spectroscopy (EIS) analysis.^13^ EIS is commonly used to study electrochemical parameters such as charge transfer and the identification of the equivalent circuit of the device, but it also represents a valuable technique for the development of impedimetric sensors.^29^

## 2 Experimental Section

### 2.1 Chemicals and Materials

All reagents including *tert*-butyl 4-aminobenzylcarbamate and solvents were purchased from Sigma-Aldrich (St. Louis, MO) and used without further purification. The CNFs (cod. PR-24-XT-PS) used in this study with an average diameter of ∼100 nm have been produced by the floating catalyst method and acquired from Pyrograf Products Inc. (Cedarville, OH). Scanning electron microscopy (SEM) images were acquired by using a Phenom ProX microscope. Thermogravimetric analyses were carried out at 10 °C/min, from 100 to 1000 °C, in an argon atmosphere using a TGA Q500 instrument (TA Instruments, New Castle, DE). Infrared spectra were obtained using a Fourier transform infrared (FTIR) Spectrum Two FTIR spectrometer (PerkinElmer Inc., Waltham, MA) by the ATR method in the range of 4000–500 cm^–1^. X-ray diffraction (XRD) analyses were performed on a Bruker D2 Phaser instrument from 10 to 90° through a PSD fast scan mode with these parameters: a step size of 0.040° and a time per step of 165 s.

### 2.2 Synthesis of CNFs-NH_2_

The synthesis of the sensitive material was carried out using carbon nanofibers (CNFs PR-24-XT-PS, supplied from Pyrograf) as follows: 500 mg of CNFs were dispersed in 1,2-dichlorobenzene (50 mL) and sonicated for 15 min. A solution of *tert*-butyl 4-aminobenzylcarbamate (1.920 g, 1.73 mmol) in acetonitrile (30 mL) was then added, and the resulting dispersion was sonicated for 30 min. Argon was bubbled into the suspension for 10 min and after the addition of isoamyl nitrite (1.75 mL, 2.6 mmol). The reaction mixture was refluxed at 60 °C for 24 h to obtain the CNFs-NH-Boc sample. After it was cooled to room temperature, the suspension was diluted with ethanol (100 mL) and filtered through a 0.1 μm Millipore membrane. The solid recovered on the filter was dispersed in ethanol (100 mL), sonicated for 30 min, filtered again, and washed with diethyl ether. The resulting solid was dried under vacuum. The protecting group was removed by treatment with 4 M HCl in dioxane to obtain sample (2); the residue was vacuum-filtered over a 0.1 μm Millipore membrane and washed thoroughly with dioxane and deionized water. The Kaiser test was used to quantify free amine groups after deprotection, taking advantage of the fact that at 120 °C, ninhydrin forms a complex that absorbs at 570 nm. The absorbance of the solution can be recorded and can be related to the concentration of free amine groups:
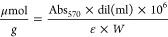
where Abs_570_ is the recorded absorbance (with a control solution with ninhydrin as the baseline), dil(ml) is the dilution (3 mL), ε is the extinction coefficient (15 000 M^–1^ cm^–1^), and *W* is the sample weight of NFs (0.2–0.4 mg). Through this test, it was possible to determine the amount of the NH_2_ group, which was found to be 471 μmol/g.

### 2.3 Synthesis of the CNFs-NH_2_/SPCE Sensor

The screen-printed carbon electrode (SPCE), modified with CNFs-NH_2_ (CNFs-NH_2_/SPCE), was prepared by dispersing 1 mg of the CNFs-NH_2_ sample in 1 mL of distilled water under sonication for 5 min. It is noteworthy that this procedure failed to effectively disperse the pure CNFs, indicating that the functionalization makes the sensor fabrication easier. Then, 10 μL of the solution was deposited on the SPCE platform and was allowed to dry at room temperature until next use.

### 2.4 Electrochemical Measurements

Electrochemical measurements were performed using screen-printed carbon electrodes purchased from Metrohm-DropSens (Metrohm Italiana S.r.l., Origgio (VA), Italy). The SPCEs consisted of a planar substrate equipped with a carbon working electrode (diameter 4 mm, geometric area 0.1257 cm^2^), a silver pseudo reference electrode and a carbon auxiliary electrode. All electrochemical analyses were performed by using a Metrohom Autolab galvanostatic potentiostat equipped with NOVA 2.1 data acquisition software. Measurements were recorded by using electrical impedance spectroscopy (EIS). EIS tests were performed using 0.1 M NaOH with a frequency range of 0.1 to 105 Hz and an amplitude of 5 mV.

## 3 Results and Discussion

### 3.1 Synthesis of Amine-Modified CNFs

**Scheme 1:**
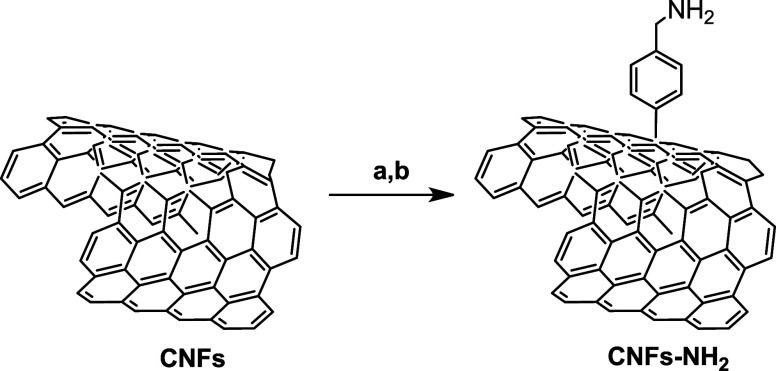
Synthesis of CNFs-NH_2_ (a) Isopentyl nitrite, *tert*-butyl 4-aminobenzylcarbamate, 1,2-dichlorobenzene, CH_3_CN, 60 °C, 24 h; (b) HCl 4 M, dioxane, 1 h, r.t.

Pyrograf-III CNFs used here have an average diameter of about 100 nm and a chemically vapor-deposited (CVD) layer of carbon on the surface of the fiber, which facilitates the surface functionalization. The synthesis of the sensitive material, CNFs-NH_2_, was then achieved by reaction of CNFs with an aryl diazonium salt containing the amine function protected with the *tert*-butyloxycarbonyl (Boc) group and generated in situ by reaction of the corresponding aniline with isopentyl nitrite, following a previously reported procedure,^30^ as depicted in Scheme 1. The amount of the free amine group, as evaluated by the Kaiser test,^31^ was found to be of 471 μmol/g.

**Figure 1:**
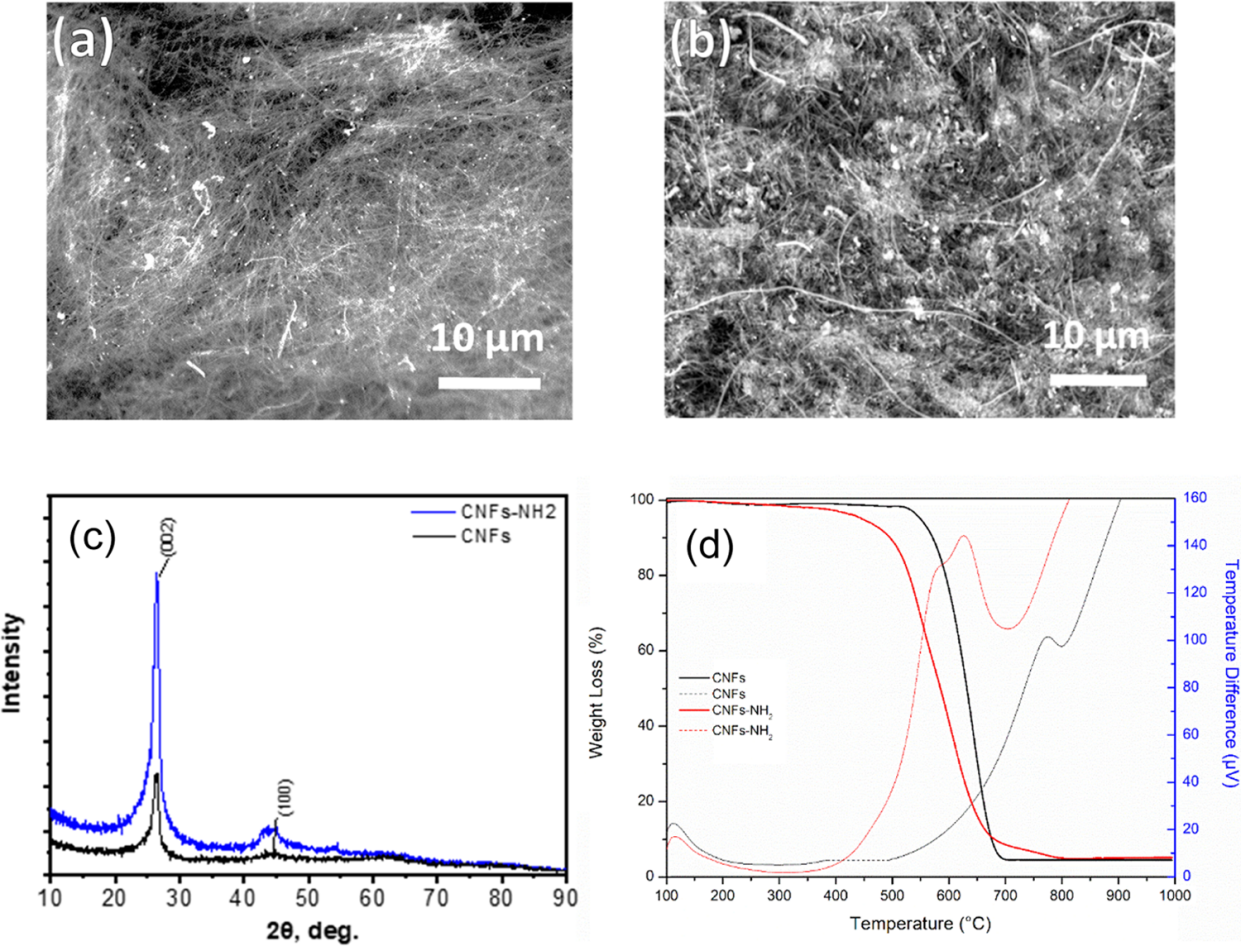
SEM images of (a) CNFs and (b) CNFs-NH_2_; (c) XRD spectra of CNFs and CNFs-NH_2_; and (d) TGA (solid lines) and differential thermal analysis (DTA) (dashed lines) profiles of CNFs and CNFs-NH_2_ performed in air.

The morphological and microstructural characteristics of the obtained material were characterized by SEM, XRD, and TGA analyses. Comparing the SEM image of pristine CNFs (Figure 1 a) with that of derived CNFs-NH_2_ (Figure 1b), it seems that the functionalization procedure does not alter the long fiber morphology of the raw carbon material.

This is also confirmed by the XRD spectra shown in Figure 1c. Indeed, in both spectra, the main peaks for both samples are at 26.61 and 44.17 °C, which indicate the C(002) and C(100) crystalline planes, respectively. The first is associated with the hexagonal structure of graphite, while the second is one of its characteristic peaks. The CNFs-NH_2_ sample was further characterized by TGA-DTA analysis performed in air while the temperature was increased at a rate of 20 °C/min up to 1000 °C to assess the thermal stability and purity (Figure 1d). For the CNFs-NH_2_ sample, the weight loss starts at a lower temperature compared to CNFs, indicating the chemical functionalization. DTA analysis also agrees with this finding, showing a well-pronounced exothermic peak at 600–640 °C, related to the decomposition of the surface NH_2_ group, while the oxidation of the CNF core is observed at a much higher temperature, leading to the complete sample’s decomposition at around 800 °C.^32^

**Figure 2:**
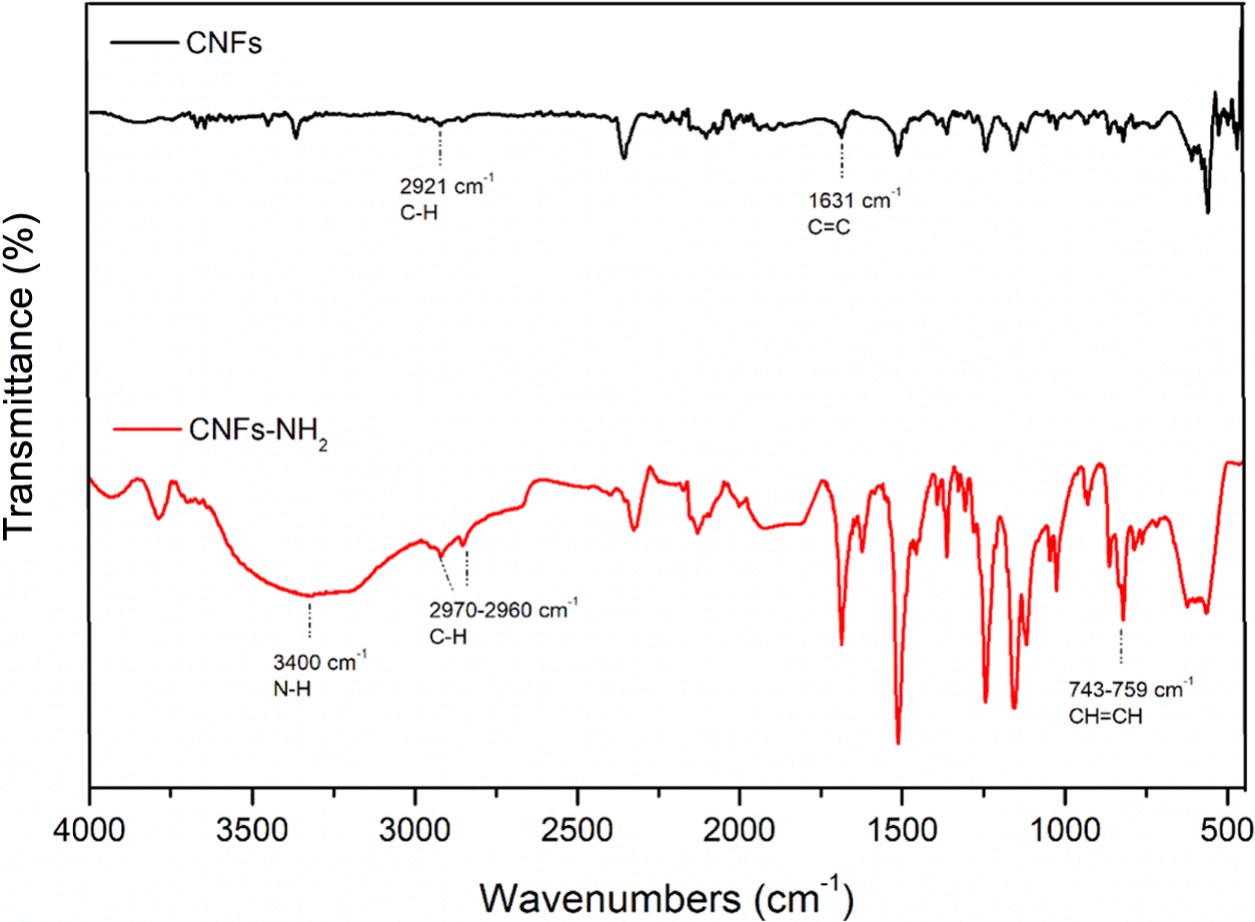
FTIR spectra of CNFs and CNFs-NH_2_.

The FTIR spectra of CNFs and of the functionalized sample (Figure 2) show, for CNFs, the C=C stretching vibration at 1631 cm^–1^ and the C–H stretching vibration at 2921 cm^–1^.

The spectrum of CNFs-NH_2_ reports the N–H stretching vibration at 3400 cm^–1^, and the peaks at about 743 and 759 cm^–1^ are related to the −CH=CH– bond vibrations of the benzene rings. In addition, a more intense peak, compared to the CNFs-NH_2_ sample, at 2970–2960 cm^–1^, can be observed due to the C–H stretching of the alkyl functionalities.

### 3.2 Electrochemical Measurements

**Figure 3:**
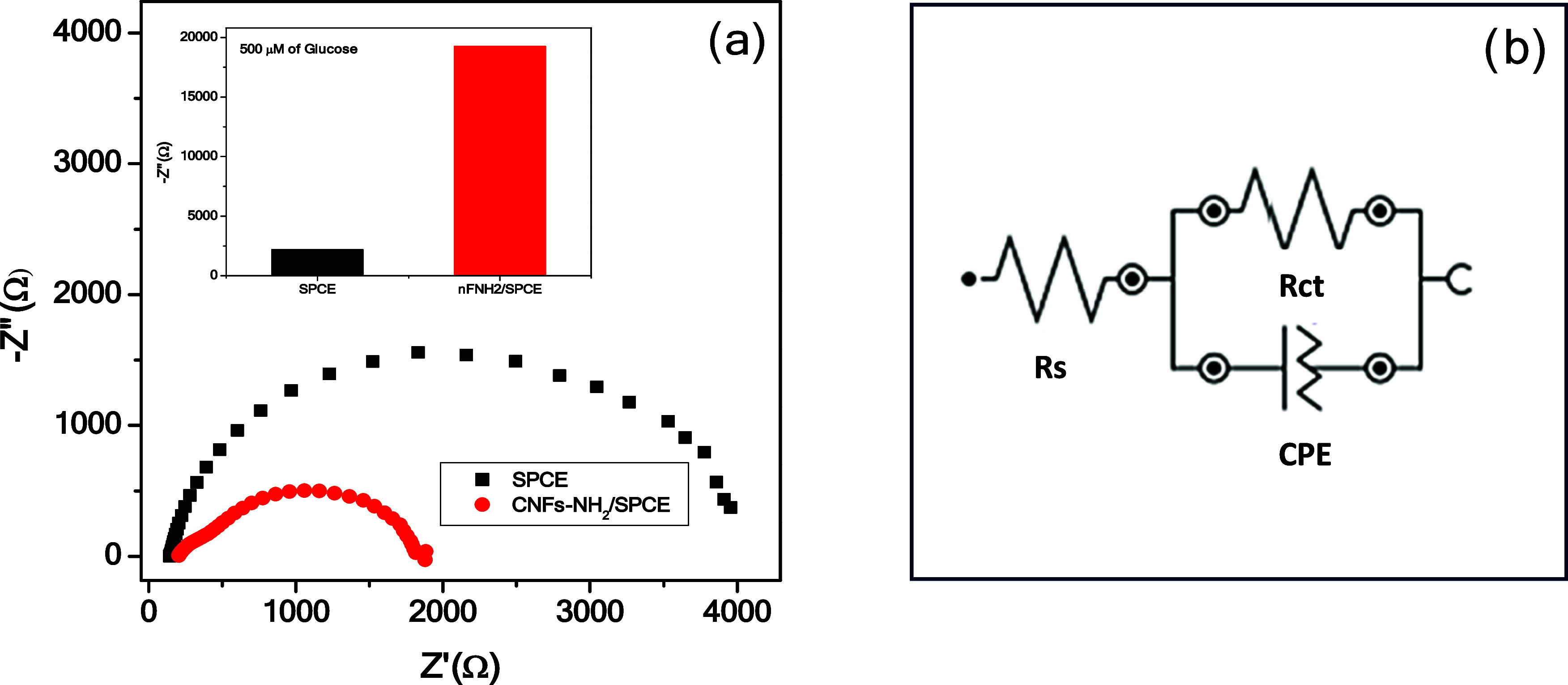
(a) EIS of the bare SPCE and CNFs-NH_2_/SPCE in a solution containing 0.1 M NaOH with a frequency range from 0.1 to 105 Hz; amplitude 5 mV. The inset shows the EIS response of the SPCE and CNFs-NH_2_/SPCE in the presence of 500 μM glucose. (b) Equivalent circuit.

The electrochemical performance of the modified CNFs-NH_2_ screen-printed carbon electrode (CNFs-NH_2_/SPCE), synthesized as reported in the experimental section, was evaluated by EIS measurements. These experiments were performed in the absence and presence of various glucose concentrations to assess the impedimetric response of the tested electrodes as electrochemical glucose sensors. The oscillation frequency was applied in the range of 100 kHz to 0.1 Hz and the set potential was applied to be 0.65 V vs Ag/AgCl. Figure 3 reports the equivalent circuit for modeling the Nyquist plot, where *R*_s_ is the electrolyte resistance, *R*_CT_ is the charge transfer resistance, and CPE is the constant phase element that models the behavior of a double layer.

**Figure 4:**
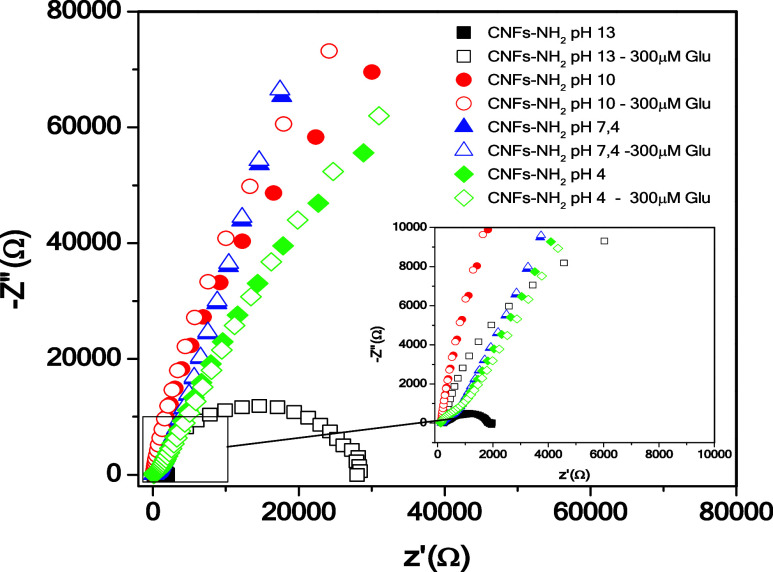
Nyquist plot for the CNFs-NH_2_/SPCE sensor in the absence and in the presence of 300 μM glucose at different pH values (pH 4, 7.4, 10, and 13).

The sensor modification was monitored by EIS analysis, comparing the curves of the modified CNFs-NH_2_/SPCE sensors with the bare one. The Nyquist plot, as represented in Figure 4, shows a large reduction of the semicircle after the CNFs-NH_2_ deposition on the working electrode surface, indicating an effective improvement of the sensor’s electrical performance by promoting charge transfer. This finding agrees with previous data reported in the literature for carbon nanomaterials.^31^ The sensor CNFs-NH_2_/SPCE was then tested and compared to the SPCE in the presence of 500 μM. The inset in Figure 4 shows the large variations of −Z″ by the CNFs-NH_2_/SPCE sensor (19 255 Ω) compared to the bare SPCE (2189 Ω).

The influence of the operating pH of the sensor is reported in Figure 4. In 0.1 M NaOH (pH = 13), there is an excellent performance of the sensor in the presence of 300 μM glucose. Indeed, a large variation of −Z″ (11 370 Ω) is noted, while as the pH decreases, the impedance variation is strongly diminished, accompanied by a remarkable change of the Nyquist plot. In neutral (pH = 7.4) and acidic (pH = 4) conditions, no impedance variation is detected.

**Figure 5:**
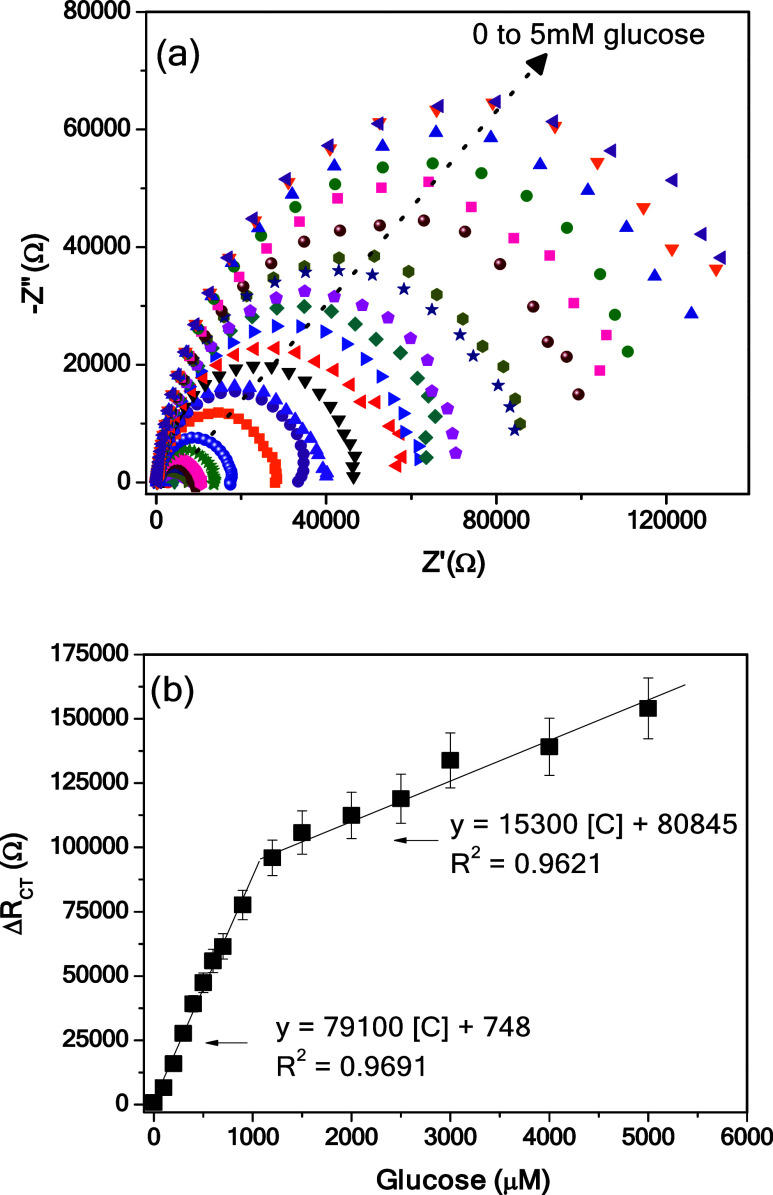
EIS analysis of the CNFs-NH_2_/SPCE sensor with variable glucose concentrations from 0 to 5000 μM in 0.1 M NaOH (pH = 13). (a) Nyquist plot and (b) calibration curve.

These results suggest the strategic role of the amino group (R-NH_2_) present on the nanofibers in the interaction with glucose to generate an impedance change. The capabilities of the CNFs-NH_2_/SPCE sensor to detect higher and variable glucose concentrations were evaluated by varying the concentration from 0 to 5000 μM in 0.1 M NaOH (pH = 13). Figure 5a shows the remarkable variation of the semicircle as the amount of glucose changes. The variation of the charge transfer resistance (Δ*R*_CT_) before and after the exposure to the different glucose concentrations, as computed by the equivalent circuit shown in Figure 3b, was used to plot the calibration curve shown in Figure 5b. The increase in *R*_CT_ with increasing glucose concentration is due to the interaction of the analyte with the amine group present on CNFs-NH_2_, leading to a modification of the electrode surface and consequently to an increase in the electron transfer resistance.

The increase in the semicircle diameter implies an enhancement in the *R*_CT_ value. Figure 5b shows the calibration curve derived from EIS analysis. Two linear trends were noted, one at low concentrations (0–1200 μM) and the other from 1200 to 5000 μM. The sensitivity (*S*) in the above linear ranges was estimated as the slope between the output signal Δ*R*_CT_ and the glucose concentration; the corresponding computed equations are reported in Figure 5b. The limit of detection (LOD) calculated as the (standard error/slope)*3.3 was 18.64 μM.

**Figure 6:**
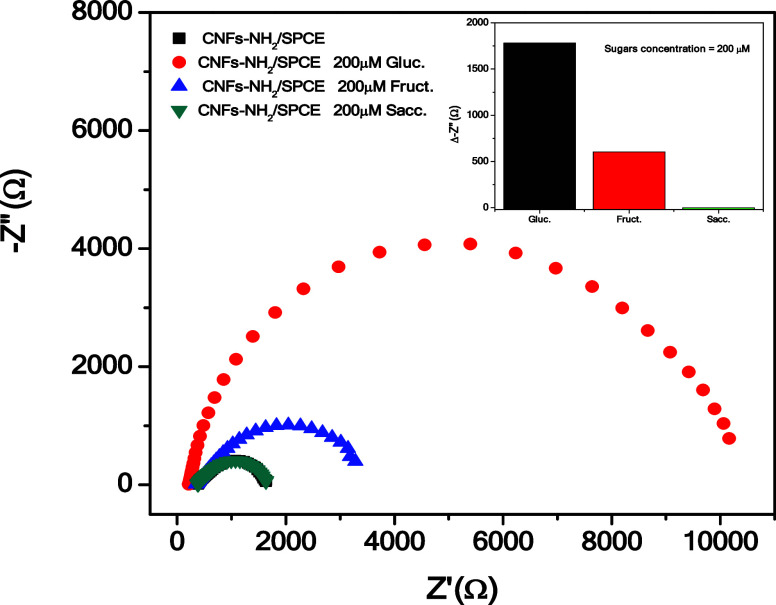
EIS analysis of the CNFs-NH_2_/SPCE sensor with 200 μM different sugars in 0.1 M NaOH (pH 13); the inset reports the response of the sensor for each sugar.

The response of the sensor to other common sugars, such as sucrose and fructose, was also investigated (Figure 6).

Interestingly, the response to the concentration of 200 μM glucose is much higher than that recorded at the same concentration with fructose; moreover, the sensor did not respond to the presence of sucrose (see the inset). This demonstrates the good ability of the CNFs-NH_2_/SPCE sensor to selectively detect the presence of glucose compared to other sugars.

### 3.3 Glucose Sensing Mechanism

**Figure 7:**
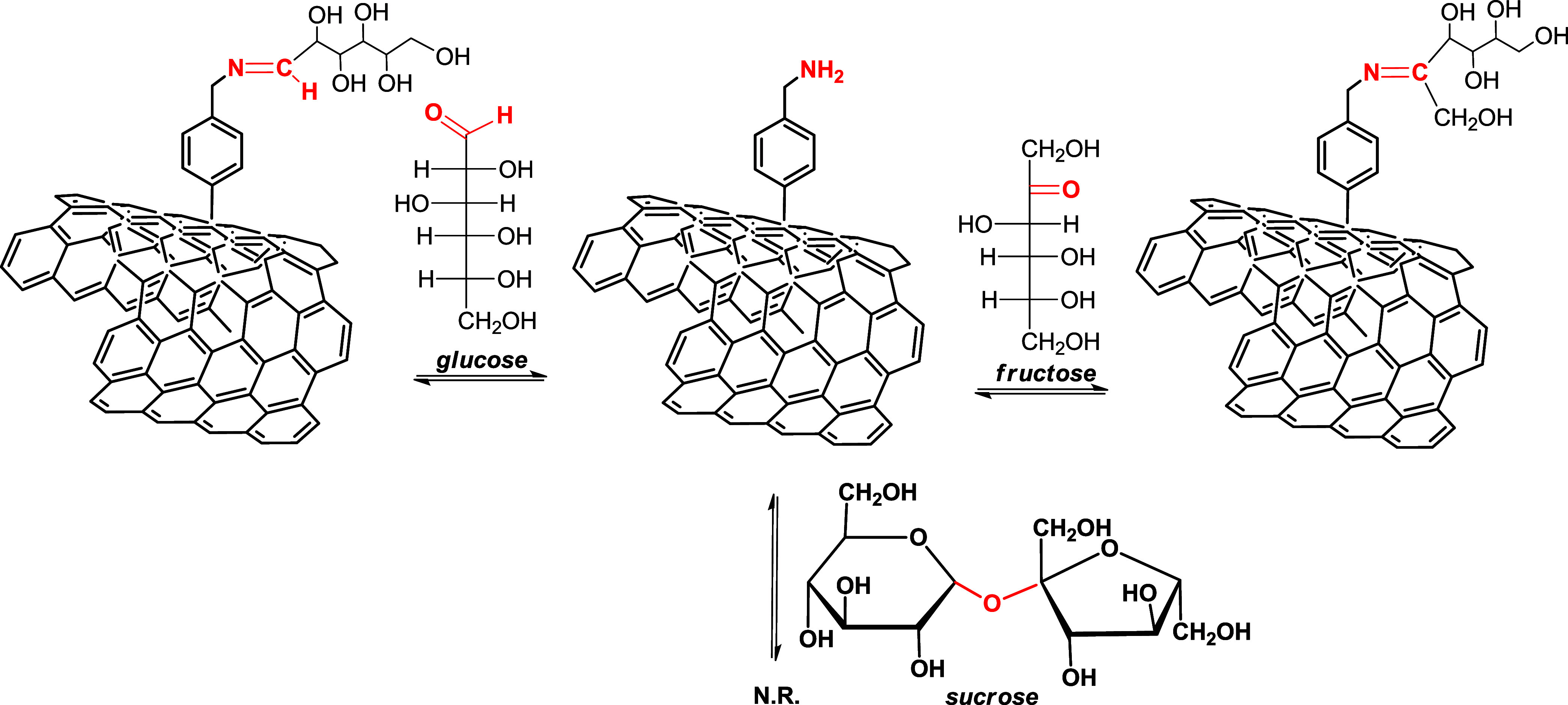
Mechanism of sugar sensing.

The observed response of tested sugars can be rationalized, considering their different chemical reactivities toward the free amino functionalities present on the electrode surface (see Figure 7). The monosaccharides, glucose and fructose, due to the presence of a free aldehyde (in glucose) or a ketone group (in fructose), are reducing sugars and can react with primary aliphatic or aromatic amines to form glycosylamines.^33^ In sucrose, no free aldehyde or ketone group is present since, in this molecule, the glycosidic bond involves the anomeric carbons of both glucose and fructose. Thus, this nonreducing sugar cannot react with amino groups to form glycosylamines.

The preferential interaction of the amino groups exposed on the surface of SPEs with glucose with respect to fructose is attributable to the higher level of reactivity of glucose with respect to the extent of glycated product formation.^34^

**Figure 8:**
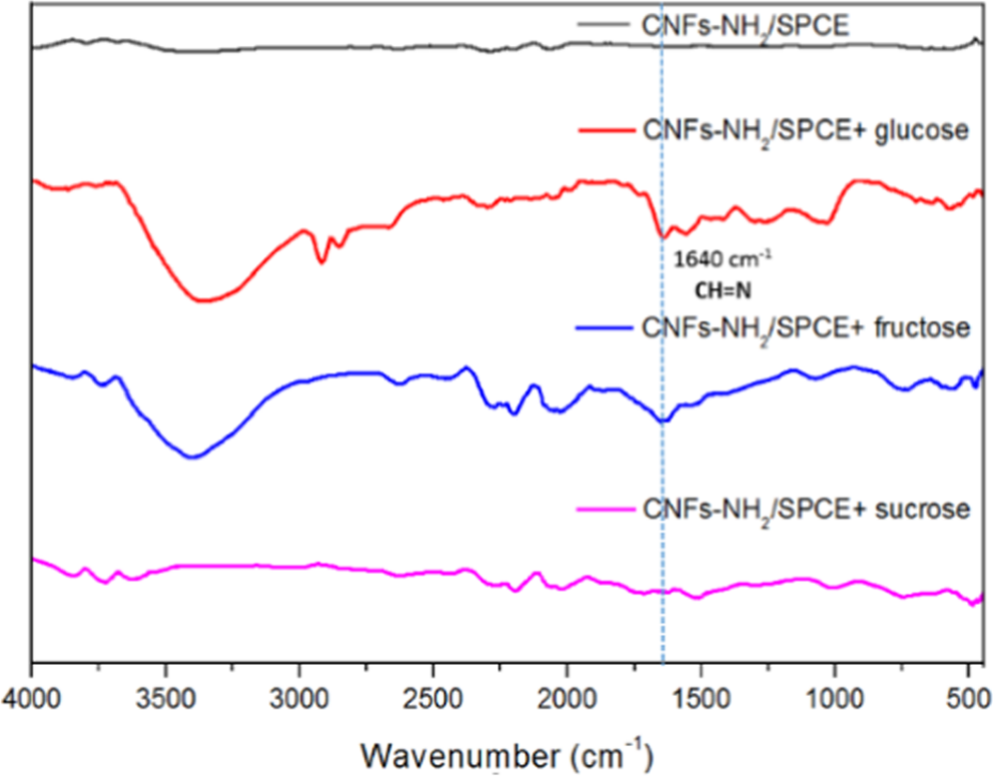
FTIR spectra of CNFs-NH_2_/SPCE, CNFs-NH_2_/SPCE + glucose, CNFs-NH_2_/SPCE + fructose,′ and CNFs-NH_2_/SPCE + sucrose after electrochemical tests.

FTIR analysis was found to be a valuable technique for investigating the glucose sensing mechanism. The preferential reaction mechanism hypothesized above was confirmed by FTIR characterization, as shown in Figure 8.

**Figure 9:**
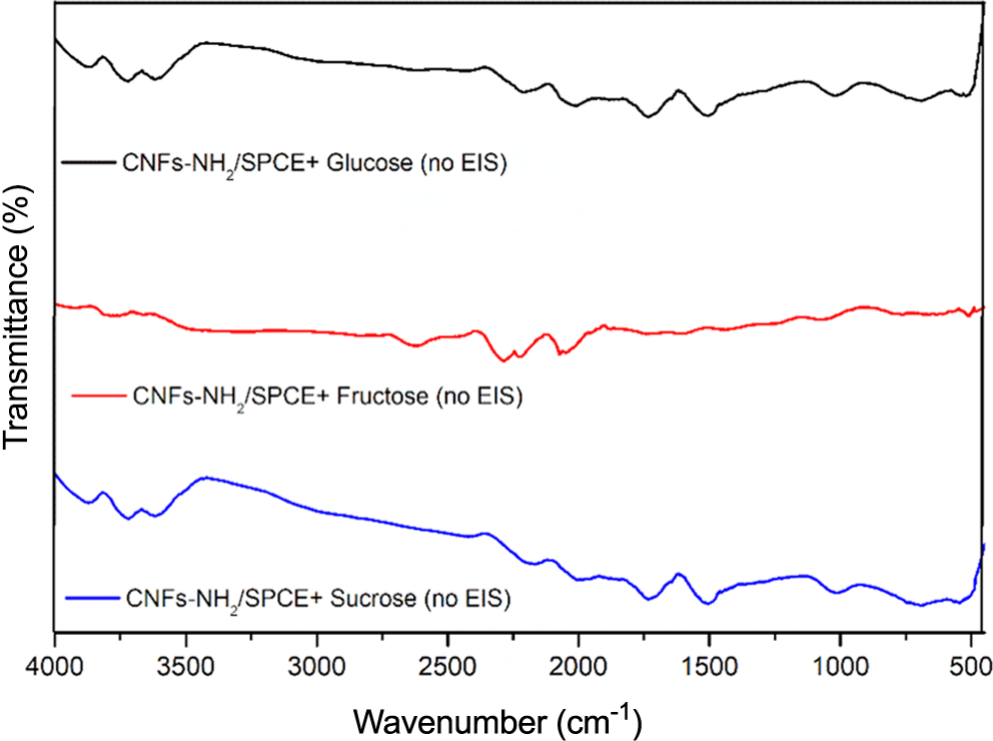
FTIR spectra of CNFs-SPCE-NH_2_/glucose, CNFs-SPCE-NH_2_/sucrose, and CNFs-SPCE-NH_2_/fructose as recorded without any potential applied.

The formation of a reversible azomethine bond between the amino group exposed on the surface of the SPCE and the carbonyl group of the sugar was observed only for glucose and fructose. The FTIR spectrum related to the interaction of the modified electrode with sucrose does not report any peaks related to this sugar. For glucose, the diagnostic peak at 1640 cm^–1^ attributable to the CH=N bond can be observed. Moreover, for this sample, the stretching at 3350 cm^–1^ due to the O–H bond, the vibrations of the C–H bonds at 2847–2915 cm^–1^, and the peak at 1040 cm^–1^ attributable to the bending vibration of the C–O bond are also recorded. The FTIR spectrum related to the interaction of fructose with the surface of CNFs-NH_2_/SPCE shows a weak peak at 1645 cm^–1^ due to the formation of the azomethine bond together with the stretching of the O–H bond at 3350 cm^–1^. The lower intensity of these peaks, with respect to that observed for glucose, agrees with the expected lower interaction of fructose with the amino groups exposed on the electrode surface. The reported EIS-promoted sensing of glucose was also demonstrated by comparing the FTIR spectra reported above with those recorded by the same substrate and sugars without applying any potential. As reported in Figure 9, no signal related to the formation of the azomethine bond can be in fact observed.

The performances of the proposed sensors such as the limit of detection (LOD), linear range, and sensitivity were compared with some recent impedimetric sensors based on the enzymatic and nonenzymatic sensing layer reported in recent literature (see Table 1). From this comparison, it appears clear that the proposed sensor displays very good performances, in terms of high sensitivity, a low detection limit, and a good linear range.

**Table 1:** Performances of the CNFs-NH_2_/SPCE Sensor for Glucose Sensing Compared to Previous Enzymatic and Nonenzymatic Impedimetric Sensors Reported in Recent Literature

electrodes	LOD (μM)	linear range (mM)	sensitivity (kΩ mM^–1^)	ref
SPCE/TiO_2_/APTES@CG/GOx	24	0.05–1	0.4	(35)
SPEs/Au/rGO	100	3.3–27–7	0.99 × 10^–3^	(36)
CE/PG/NF/FC/GOx	21	1–15	10.6	(37)
CNFs-NH_2_/SPCE	18.6	0–5	79.1	this work

## 4 Conclusions

A simple wet chemistry functionalization of carbon nanofibers with an aromatic diamine provided a sensing material for the development of a high-performance enzyme-free electrochemical sensor for the selective sensing of glucose. The electrochemical performance of the modified carbon-based screen-printed electrode was evaluated by EIS, demonstrating a high selectivity of the sensor for glucose with respect to fructose and sucrose. The optimization of the sensing parameters allowed us to obtain linear calibrations at low (0–1200 mM) and high (1200–5000 mM) glucose concentrations and a low (18.64 mM) LOD. A selective glucose sensing mechanism on the novel electrodes has been proposed based on the formation of a reversible azomethine bond between the amino group exposed on the surface of CNFs-NH_2_/SPCE and the carbonyl group of the sugar. The results of this study highlight the performance of this novel electrochemical sensor for the selective sensing of this biologically relevant analyte. Further, the EIS technique used is suitable for future glucose monitoring in a continuous manner. The proposed sensor also has the advantage of being a nonenzymatic sensor, allowing its use in extreme working environments where enzymatic sensors could not operate, such as in analysis of fermentation processes,^38^ and it does not need any redox probe.

## References

[ref1] Galant A. L.; Kaufman R. C.; Wilson J. D. Glucose: Detection and analysis. Food Chem. 2015, 188, 149–160. 10.1016/j.foodchem.2015.04.071. 26041177

[ref2] Jarvis P. R. E.; Cardin J. L.; Nisevich-Bede P. M.; McCarter J. P. Continuous glucose monitoring in a healthy population: understanding the post-prandial glycemic response in individuals without diabetes mellitus. Metabolism 2023, 146, 155640 10.1016/j.metabol.2023.155640. 37356796

[ref3] Xue Y.; Thalmayer A. S.; Zeising S.; Fischer G.; Lübke M. Commercial and Scientific Solutions for Blood Glucose Monitoring—A Review. Sensors 2022, 22 (2), 425 10.3390/s22020425. 35062385 PMC8780031

[ref4] Tomic D.; Shaw J. E.; Magliano D. J. The burden and risks of emerging complications of diabetes mellitus. Nat. Rev. Endocrinol. 2022, 18, 525–539. 10.1038/s41574-022-00690-7. 35668219 PMC9169030

[ref5] Wang B. T.; Hu S.; Yu X. Y.; Jin L.; Zhu Y. J.; Jin F. J. Studies of Cellulose and Starch Utilization and the Regulatory Mechanisms of Related Enzymes in Fungi. Polymers 2020, 12 (3), 530 10.3390/polym12030530. 32121667 PMC7182937

[ref6] Goldrick S.; Lee K.; Spencer C.; Holmes W.; Kuiper M.; Turner R.; Farid S. S. On-Line Control of Glucose Concentration in High-Yielding Mammalian Cell Cultures Enabled Through Oxygen Transfer Rate Measurements. Biotechnol. J. 2018, 13 (4), 1700607 10.1002/biot.201700607. 29247603

[ref7] Wang X.; Luo Y.; Huang K.; Cheng N. Biosensor for agriculture and food safety: Recent advances and future perspectives. Adv. Agrochem. 2022, 1 (1), 3–6. 10.1016/j.aac.2022.08.002.

[ref8] Huang H.; Qian M.; Gao Q.; Zhang C.; Qi H. A sensitive and noninvasive cyclic peptide-based electrogenerated chemiluminescence biosensing method for the determination of sweat glucose. Chem. Commun. 2023, 59, 8941–8944. 10.1039/D3CC02549G. 37394953

[ref9] Wei M.; Qiao Y.; Zhao H.; Liang J.; Li T.; Luo Y.; Lu S.; Shi X.; Lu W.; Sun X. Electrochemical non-enzymatic glucose sensors: recent progress and perspectives. Chem. Commun. 2020, 56, 14553–14569. 10.1039/D0CC05650B. 33118566

[ref10] Tiwari C.; Jha S. S.; Kumar R.; Chhabra M.; Malhotra B. D.; Dixit A. Exfoliated graphite carbon paper-based flexible nonenzymatic glucose sensor. Mater. Sci. Eng., B 2022, 285, 115931 10.1016/j.mseb.2022.115931.

[ref11] Namkoong Y.; Oh J.; Hong J. I. Electrochemiluminescent detection of glucose in human serum by BODIPY-based chemodosimeters for hydrogen peroxide using accelerated self-immolation of boronates. Chem. Commun. 2020, 56, 7577–7580. 10.1039/D0CC03315D. 32510098

[ref12] Gnana kumar G.; Amala G.; Gowtham S. M. Recent advancements, key challenges and solutions in non-enzymatic electrochemical glucose sensors based on graphene platforms. RSC Adv. 2017, 7, 36949–36976. 10.1039/C7RA02845H.

[ref13] Huang J.; Zhang Y.; Wu J. Review of non-invasive continuous glucose monitoring based on impedance spectroscopy. Sens. Actuators, A 2020, 311, 112103 10.1016/j.sna.2020.112103.

[ref14] Wikeley S. M.; Przybylowski J. P.; Lozano-Sanchez M.; Caffio T. D.; James S. D.; Bull; Fletcher P. J.; Marken F. Polymer indicator displacement assay: electrochemical glucose monitoring based on boronic acid receptors and graphene foam competitively binding with poly-nordihydroguaiaretic acid. Analyst 2022, 147, 661–670. 10.1039/D1AN01991K. 35060574

[ref15] Acevedo-Restrepo I.; Blandón-Naranjo L.; Hoyos-Arbeláez J.; Della Pelle F.; Vázquez M. V. Electrochemical Glucose Quantification as a Strategy for Ethanolic Fermentation Monitoring. Chemosensors 2019, 7, 14 10.3390/chemosensors7010014.

[ref16] Gupta P.; Gupta V. K.; Huseinov A.; Rahm C. E.; Gazica K.; Alvarez N. T. Highly sensitive non-enzymatic glucose sensor based on carbon nanotube microelectrode set. Sens. Actuators B Chem. 2021, 348 (1), 130688 10.1016/j.snb.2021.130688.

[ref17] Mohammadpour-Haratbar A.; Mohammadpour-Haratbar S.; Zare Y.; Rhee K. Y.; Park S. J. A Review on Non-Enzymatic Electrochemical Biosensors of Glucose Using Carbon Nanofiber Nanocomposites. Biosensors 2022, 12, 1004 10.3390/bios12111004. 36421123 PMC9688744

[ref18] Iannazzo D.; Celesti C.; Espro C.; Ferlazzo A.; Giofrè S. V.; Scuderi M.; Scalese S.; Gabriele B.; Mancuso R.; Ziccarelli I.; et al. Orange-Peel-Derived Nanobiochar for Targeted Cancer Therapy. Pharmaceutics 2022, 14, 2249 10.3390/pharmaceutics14102249. 36297682 PMC9607014

[ref19] Phetsang S.; Kidkhunthod P.; Chanlek N.; Jakmunee J.; Mungkornasawakul P.; Ounnunkad K. al. Copper/reduced graphene oxide film modified electrode for non-enzymatic glucose sensing application. Sci. Rep. 2021, 11, 9302 10.1038/s41598-021-88747-x. 33927300 PMC8085015

[ref20] Tam T. V.; Hur S. H.; Chung J. S.; Choi W. M. Novel paper- and fiber optic-based fluorescent sensor for glucose detection using aniline-functionalized graphene quantum dots. Sens. Actuators B Chem. 2021, 329, 129250 10.1016/j.snb.2020.129250.

[ref21] Bressi V.; Chiarotto I.; Ferlazzo A.; Celesti C.; Michenzi C.; Len T.; Iannazzo D.; Neri G.; Espro C. Voltammetric Sensor Based on Waste-Derived Carbon Nanodots for Enhanced Detection of Nitrobenzene. ChemElectroChem 2023, 10, e202300 10.1002/celc.202300004.

[ref22] Wu K.-L.; Cai Y.-.M.; Jiang B.-.B.; Cheong W.-.C.; Wei X.-.W.; Wang W.; Yu N. Cu@Ni core–shell nanoparticles/reduced graphene oxide nanocomposites for nonenzymatic glucose sensor. RSC Adv. 2017, 7, 21128–21135. 10.1039/C7RA00910K.

[ref23] Abid K.; Foti A.; Khaskhoussi A.; Celesti C.; D’Andrea C.; Polykretis P.; Matteini P.; Iannazzo D.; Maalej R.; Gucciardi P. G.; Neri G. A study of screen-printed electrodes modified with MoSe2 and AuNPs-MoSe2 nanosheets for dopamine sensing. Electrochim. Acta 2024, 475, 143371 10.1016/j.electacta.2023.143371.

[ref24] Othman H. O.; Hassan R. O.; Faizullah A. T. A newly synthesized boronic acid-functionalized sulfur-doped carbon dot chemosensor as a molecular probe for glucose sensing. Microchem. J. 2021, 163, 105919 10.1016/j.microc.2021.105919.

[ref25] Lerner M. B.; Kybert N.; Mendoza R.; Villechenon R.; Lopez M. A. B.; Johnson C. Scalable, non-invasive glucose sensor based on boronic acid functionalized carbon nanotube transistors. Appl. Phys. Lett. 2013, 102, 183113 10.1063/1.4804438.

[ref26] Stavyiannoudaki V.; Vamvakaki V.; Chaniotakis N. Comparison of protein immobilisation methods onto oxidised and native carbon nanofibres for optimum biosensor development. Anal. Bioanal. Chem. 2009, 395, 429–435. 10.1007/s00216-009-2970-y. 19644678

[ref27] Wang Z.; Wu S.; Wang J.; Yu A.; Wei G. Carbon Nanofiber-Based Functional Nanomaterials for Sensor Applications. Nanomaterials 2019, 9, 1045 10.3390/nano9071045. 31336563 PMC6669495

[ref28] Klein K. L.; Melechko A. V.; McKnight T. E.; Retterer S. T.; Rack P. D.; Fowlkes J. D.; Joy D. C.; Simpson M. L. Surface characterization and functionalization of carbon nanofibers. J. Appl. Phys. 2008, 103, 061301 10.1063/1.2840049.

[ref29] Loew N.; Watanabe H.; Shitanda I.; Itagaki M. Electrochemical impedance spectroscopy: Simultaneous detection of different diffusion behaviors as seen in finite element method simulations of mediator-type enzyme electrodes. Electrochim. Acta 2022, 421, 140467 10.1016/j.electacta.2022.140467.

[ref30] Iannazzo D.; Pistone A.; Galvagno S.; Ferro S.; De Luca L.; Monforte A. M.; Da Ros T.; Hadad C.; Prato M.; Pannecouque C. Synthesis and anti-HIV activity of carboxylated and drug-conjugated multi-walled carbon nanotubes. Carbon 2015, 82, 548–561. 10.1016/j.carbon.2014.11.007.

[ref31] Sarin V. K.; Kent S. B. H.; Tam J. P.; Merrifield R. B. Quantitative monitoring of solid-phase peptide synthesis by the ninhydrin reaction. Anal. Biochem. 1981, 117, 147–157. 10.1016/0003-2697(81)90704-1. 7316187

[ref32] Jang H.-N.; Jo M.-H.; Ahn H.-J. Tailored functional group vitalization on mesoporous carbon nanofibers for ultrafast electrochemical capacitors. Appl. Surf. Sci. 2023, 623, 157081 10.1016/j.apsusc.2023.157081.

[ref33] Wang S. Z.; Zhen M.; Xue Z.; Zhuo M. P.; Zhou Q.; Su Y.; Zheng M.; Yuan G.; Wang Z. S. Flowerlike CuO/Au Nanoparticle Heterostructures for Nonenzymatic Glucose Detection. ACS Appl. Nano Mater. 2021, 4 (6), 5808–5815. 10.1021/acsanm.1c00607.

[ref34] Gokhale M. Y.; Kearney W. R.; Kirsch L. E. Glycosylation of Aromatic Amines I: Characterization of Reaction Products and Kinetic Scheme. AAPS Pharm. Sci. Tech 2009, 10, 317–328. 10.1208/s12249-009-9209-2. PMC2690773 19306062

[ref35] Ognjanović M.; Stanković V.; Knežević S.; Antić B.; Vranješ-Djurić S.; Stanković D. M. TiO_2_/APTES cross-linked to carboxylic graphene based impedimetric glucose biosensor. Microchem. J. 2020, 158, 105150 10.1016/j.microc.2020.105150.

[ref36] Ahmadi A.; Khoshfetrat S. M.; Kabiri S.; Fotouhi L.; Dorraji P. S.; Omidfar K. Impedimetric paper-based enzymatic biosensor using electrospun cellulose acetate nanofiber and reduced graphene oxide for detection of glucose from whole blood. IEEE Sens. J. 2021, 21, 9210–9217. 10.1109/JSEN.2021.3053033.

[ref37] Zarei A.; Hatefi-Mehrjardi A.; Ali Karimi M.; Mohadesi A. Impedimetric glucose biosensing based on drop-cast of porous graphene, nafion, ferrocene, and glucose oxidase biocomposite optimized by central composite design. J. Electroanal. Chem. 2022, 919, 116544 10.1016/j.jelechem.2022.116544.

[ref38] Espro C.; S Marini S.; Giusi D.; Ampelli C.; Neri G. Non-enzymatic screen printed sensor based on Cu2O nanocubes for glucose determination in bio-fermentation processes. J. Electroanal. Chem. 2020, 873, 114354 10.1016/j.jelechem.2020.114354.

